# Objective Analysis of Traditional Chinese Medicine Syndrome Differentiation of Patients With Diabetes and Prediabetes: Protocol for a Nonrandomized, Exploratory, Observational Case-Control Study Using Digitalized Traditional Chinese Medicine Diagnostic Tools

**DOI:** 10.2196/56024

**Published:** 2024-09-12

**Authors:** Hui Ping Ng, Shu Yun Chong, Yi Huan Li, Tong Hwee Goh, Ka Yii Pang, Michelle Jessica Pereira, Chin-Ming Huang

**Affiliations:** 1 Singapore Chung Hwa Medical Institution Singapore Singapore; 2 Singapore College of Traditional Chinese Medicine Singapore Singapore; 3 Health Services and Outcomes Research (HSOR) National Healthcare Group Singapore Singapore; 4 School of Post-Baccalaureate Chinese Medicine China Medical University Taichung Taiwan

**Keywords:** diabetes, dyslipidemia, hypertension, prediabetes, traditional Chinese medicine syndrome differentiation, pulse diagnosis, tongue diagnosis, tongue, diabetic, protocol, diagnostic tools, diagnostic system, accuracy, diagnosis, treatment, prevention, questionnaire, sugar intake, physical activity, health evaluation

## Abstract

**Background:**

Diabetes and prediabetes are diagnosed differentially by Western and Chinese medicine. While Western medicine uses objective laboratory analysis of biochemical parameters to define the severity of diabetes and prediabetes, Chinese medicine uses a comprehensive approach that integrates observation, inquiry, pulse palpation, and tongue diagnosis. The medical information collected is then categorized into different syndromes. However, traditional methods of pulse and tongue diagnoses used to determine syndrome differentiation are highly subjective and skill dependent.

**Objective:**

This study aims to identify the gap in conventional traditional Chinese medicine (TCM) diagnostic techniques for syndrome differentiation analysis using contemporary diagnostic devices. We devised a protocol for a nonrandomized, exploratory, observational case-control study with equal allocations in 5 arms to investigate the syndrome differentiation of diabetes and prediabetes. We hypothesize that the TCM syndrome differentiation of diabetes and prediabetes in the tropical climate may differ from that defined based on the Chinese demographic. We also speculate that the high-frequency spectral energy may reflect a difference in pulse wave intensity and density between the healthy and diabetes groups.

**Methods:**

A total of 250 eligible participants will be equally assigned to 1 of 5 arms (healthy or subhealthy, prediabetes, diabetes, prediabetes with hypertension and dyslipidemia, and diabetes with hypertension and dyslipidemia). Participants aged 21-75 years, of any sex or race, and have been diagnosed with diabetes (fasting plasma glucose [FPG] of 7 mmol/L, or 2-hour plasma glucose [2hPG] of 11.1 mmol/L) or prediabetes (impaired FPG of 6.1-6.9 mmol/L, or impaired glucose tolerance with an 2hPG of 7.8-11 mmol/L) will be included. The Health Evaluation Questionnaire, Physical Activity Questionnaire, sugar intake assessment, Constitution in Chinese Medicine Questionnaire, radial pulse diagnosis, and tongue diagnosis will be performed in a single visit. ANOVA for continuous data and chi-square tests of independence will be used for categorical data assessments, with a level of *P*<.05 considered significant.

**Results:**

The recruitment is in progress. We anticipate that the study will conclude in June 2025. As of July 15, 2024, we have enrolled 140 individuals.

**Conclusions:**

To the best of our knowledge, this is the first study to use contemporary TCM diagnostic instruments to map expert and empirical knowledge of TCM to its scientific equivalents for the purpose of evaluating the syndrome differentiation of diabetes. We designed this protocol with the exploratory goal to examine objectively the syndrome differentiation of patients with diabetes and those with prediabetes using TCM diagnostic technologies. The data collected and evaluated under standardized conditions using these contemporary diagnostic devices will exhibit a higher degree of stability, hence yielding dependable and unbiased results for syndrome differentiation. Thus, our findings may potentially increase the accuracy of identification, diagnosis, treatment, and prevention of diabetes and prediabetes through a system of targeted treatment.

**Trial Registration:**

ClinicalTrials.gov NCT05563090; https://clinicaltrials.gov/ct2/show/NCT05563090

**International Registered Report Identifier (IRRID):**

DERR1-10.2196/56024

## Introduction

### Definition

Diabetes mellitus is one of the top 10 leading causes of mortality, presenting a significant burden on global public health [1]. The International Diabetes Federation estimates that the global diabetes prevalence among individuals aged 20-79 years will increase from 10.5% (536.6 million) to 12.2% (783.2 million) in 2045 [2]. Prediabetes (intermediate hyperglycemia), characterized by a glycemic level between normal glucose tolerance and diabetes, maintains a high risk of developing diabetes, with an annual conversion rate of 5%-10% [3]. Table 1 provides the World Health Organization (WHO) diagnostic standards defined for diabetes and prediabetes [4]. The American Diabetes Association also states that the glycated hemoglobin A_1c_ (HbA_1c_) level should be at or above 6.5% for diabetes and between 5.7% and 6.4% for prediabetes [5]. The projected global prevalence of patients with impaired fasting glucose and impaired glucose tolerance is 6.5% (414 million) and 10.0% (638 million), respectively [5].

**Table 1:** The WHO^a^ definition of diabetes and prediabetes.

Type and test				WHO criteria		
**Diabetes**						
	FPG^b^			≥7.0		126
	2hPG^c^			≥11.1		200
**Intermediate hyperglycemia (prediabetes)**						
	**IFG^d^**					
		FPG	6.1-6.9		110-125	
		2hPG	<7.8		140	
	**IGT^e^**					
		FPG	<7.0		126	
		2hPG	7.8-11.1		140-200	

^a^WHO: World Health Organization.

^b^FPG: fasting plasma glucose.

^c^2hPG: 2-hour plasma glucose.

^d^IFG: impaired fasting glucose.

^e^IGT: impaired glucose tolerance.

### Prevalence of Diabetes and Prediabetes in Singapore

A local study concluded that between 2010 and 2035, the prevalence of prediabetes and diabetes among Singapore residents is expected to increase by more than 2-fold, from 434,685 to 903,596 and from 373,104 to 823,802, respectively. The incidence of prediabetes and diabetes is respectively projected to increase gradually from 15.5% to 24.9% and from 13.3% to 22.7% [6]. The number of patients with diabetes in Singapore is projected to grow to 1,000,000 in 2050 [7]. It is noted that Indians have the highest prevalence of diabetes in Singapore at 17.2%, followed closely by Malays at 16.6%, and Chinese at 9.7% [8].

### Progression of the Disease

Prediabetics have a 5%-10% chance of progressing to diabetes and a higher risk of developing complications such as early nephropathy, small fiber neuropathy, early retinopathy, and risk of macrovascular disease [3,9,10].

Adults with diabetes have double the chance of contracting heart disease and stroke [11,12]. Patients with diabetes are also prone to developing kidney failure and blindness from diabetic retinopathy [13,14]. Hyperglycemia could cause neuropathy, foot ulcers, and limb amputation [15,16]. In 2021, the proportion of diabetes-related deaths in Singapore under the age of 60 years was 31.4% [17]. In addition, the total diabetes-related health expenditure worldwide in 2021 amounts to US $966 billion [17]. In Singapore, 11% of the total health care budget is set aside for diabetes [18]. It is to be noted that diabetes tends to hinder employment and productivity, putting more stress on the economy [19,20].

### Different Diagnostic Approaches From Western and Chinese Medicines

The diagnosis of diabetes and prediabetes in Western and Chinese medicines differs in the approach, methodology, and fundamental principle. Objective laboratory analysis of biochemical parameters such as fasting plasma glucose (FPG), HbA_1c_, and oral glucose tolerance tests play a crucial role in the diagnosis of diabetes in Western medicine [21]. In addition, salivary biochemical analysis is also used as a diagnostic tool for diabetes [22,23]. Diabetes and prediabetes result from inadequate insulin secretion or insulin resistance.

In contrast to Western medicine diagnostic criteria, traditional Chinese medicine (TCM) uses a different approach in the diagnosis of diabetes and prediabetes. TCM uses a unique diagnostic system to determine the underlying causes of the symptoms and devise individualized treatment plans to restore balance and promote health. Conventionally, TCM practitioners examine the patient’s body appearance, tongue and radial pulse characteristics, medical history, and symptoms to provide a comprehensive and holistic analysis of the disease syndrome. The syndrome differentiation is an assemblage of clinical manifestations resulting from the disharmony or imbalances of the body’s yin and yang, qi, and blood [24].

The ancient Chinese name of diabetes in the TCM classic “*Huang Di Nei Jing*” is known as “*xiao-ke*” [25,26]. Table 2 summarizes the syndrome differentiation of “*xiao-ke*” in the current *TCM Internal Medicine Textbook* [27].

**Table 2:** Classification of “xiao-ke (消渴)” based on syndrome-etiology differentiation.

Type of syndrome and classification			Main clinical manifestations		Other symptoms
***shang-xiao*** **上消**					
	Excess lung heat and fluid deficiency 肺热津伤	Polydipsia		Dry mouth and tongue, frequent polyuria, excessive sweating with dysphoria. The tongue tip is red with a thin yellow coating. Surging and rapid pulse.	
***zhong-xiao*** **中消**					
	Excess stomach heat 胃热炽盛	Polyphagia, weight loss		Thirst, excessive urine, dry stool. Yellow tongue coating. Slippery and forceful pulse.	
	Qi and yin deficiency 气阴亏虚	Polydipsia		Diarrhea, with normal appetite or poor appetite, lassitude, weak limbs, and weight loss.	
***xia-xiao*** **下消**					
	Kidney yin deficiency 肾阴亏虚	Polyuria		Excessive, turbid, sweet urine, weak and sore back and knees, fatigue, dizziness, tinnitus, dry mouth and tongue, dry skin, skin itch. Red tongue with faint coating. Fine and rapid pulse.	
	Yin and yang deficiency 阴阳两虚	Polyuria		Frequent turbid urination may even pass urine upon water intake, haggard complexion, dry ears, weak and sore back and knees, cold limbs, aversion to cold, impotence, or irregular menstruation. Pale, white, and dry tongue. Sunken, fine, and weak pulse.	

TCM provides individualized treatment plans based on the syndrome differentiation and body constitution to address the root causes of illness and promote overall well-being.

Comparing the Western medicine and TCM diagnostic approaches in diabetes and prediabetes highlights their complementary strengths. Western medicine excels in precise diagnosis and blood sugar management, while TCM offers a holistic perspective focusing on underlying imbalances. Integrating these approaches could offer a more comprehensive approach to managing diabetes and prediabetes.

### Current Possible Gaps in Syndrome Classification of Diseases

It should be noted that the syndrome classification was developed using China’s demographic data. We speculatively believe that this could be notably dissimilar from countries with tropical climates like Singapore due to the region’s specific geographical and meteorological characteristics. Local research on the body constitution of a multiracial population revealed that qi-deficiency, phlegm dampness, and damp heat were the 3 most prevalent conditions [28]. Another local study reported a significant difference in the syndrome differentiation of knee osteoarthritis, wherein a much higher proportion (51%) of these patients experience spleen and kidney deficiencies with dampness syndrome compared to only 4.2% of those in China [29].

### Conventional TCM Diagnostic Techniques

**Figure 1:**
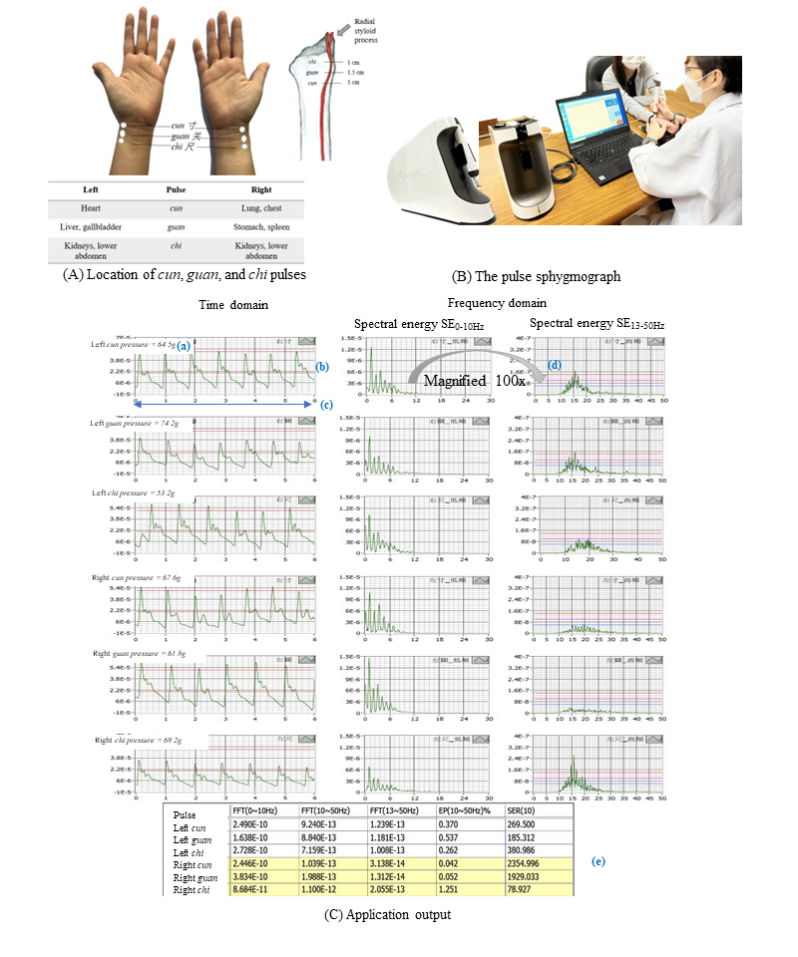
(A) Location of cun, guan, and chi pulses on the radial artery. (B) Modern pulse sphygmograph and its application output in graphical and quantitative measures. (C) Application output indicating (a) palpating pressure; (b) pulse wave; (c) pulse rate per 6 seconds; (d) intensity and density of the wave spectral energy; and (e) quantitative measures of spectral energy, energy proportion, and spectral energy ratio.

Moreover, the conventional techniques of diagnosis which rely solely on the clinical judgment, finger sensitivity, and visual accuracy of the TCM practitioner can result in inconsistent diagnoses and treatment [30,31]. For instance, the practitioner uses the index, middle, and ring fingers to simultaneously or separately palpate the bilateral radial pulses at *cun* (distal), *guan* (middle), and *chi* (proximal) locations corresponding to each viscus on each wrist (Figure 1) [31].

Historical TCM references list 28 common pulse profiles seen in clinical practice [32]. Each radial pulse profile, with its distinct characteristics in the pulse depth, rate, waveform, density, and intensity, can reveal physiological or pathological changes in the human body [33]. For instance, Jeon et al [34] investigated pulse wave variation during the female menstrual cycle and reported that string-like or slippery and rapid pulses were seen. Subjective evaluations have been demonstrated to influence the diagnosis and treatments [31].

**Figure 2:**
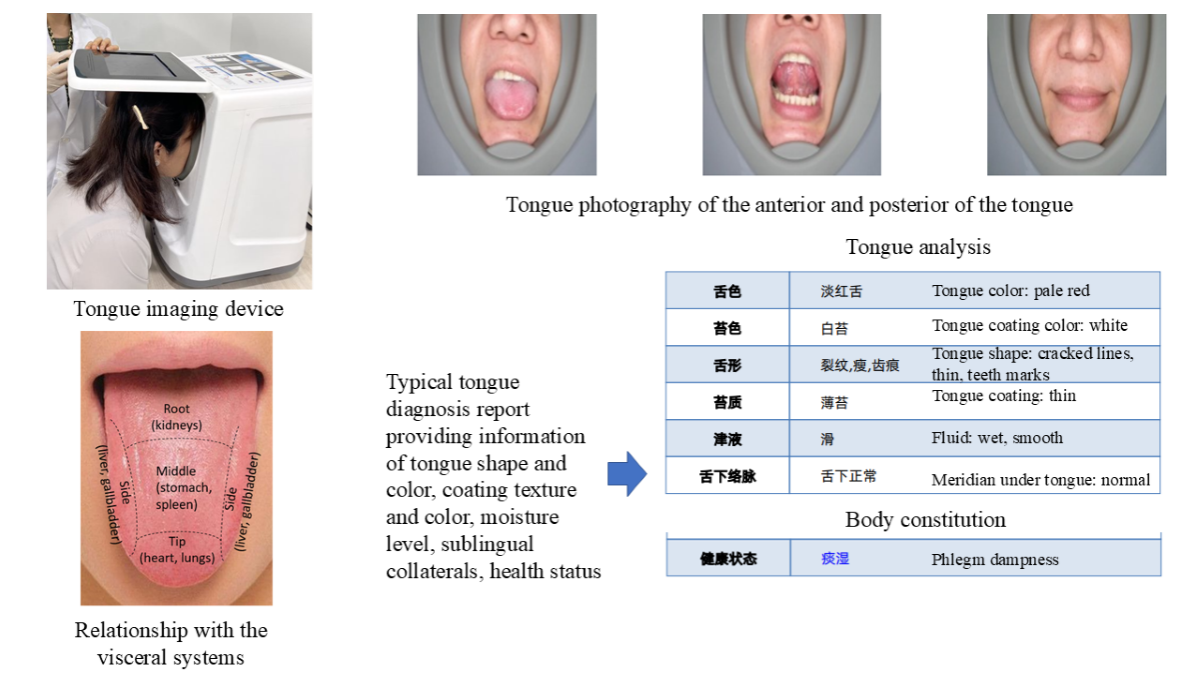
Relationship of the tongue and the respective viscera in TCM concept and tongue imaging analysis. TCM: traditional Chinese medicine.

Aside from pulse diagnosis, tongue diagnosis is essential in distinguishing TCM syndromes. The practitioner examines the physiological and pathological changes in the tongue and associates them with specific viscera. Figure 2 provides the relationship between the tongue position and the viscera (*zang-fu*) systems. However, because observations are based on the physician’s judgment and can be easily influenced by environmental factors such as lighting and colored food, they can be highly subjective and inaccurate [35].

### Specific Aims and Hypothesis

This study aims to identify the gap in syndrome differentiation between the subjective and objective TCM diagnosis. We devised a protocol for a nonrandomized, exploratory, observational case-control study with equal allocations in 5 arms to investigate the syndrome differentiation of diabetes and prediabetes. We hypothesize that the local TCM syndrome differentiation of diabetes and prediabetes in the tropical climate may differ from that defined based on the Chinese demographic. We also speculate that the high-frequency spectral energy (SE_13-50Hz_) may reflect a difference in pulse wave intensity and density between the healthy and diabetes groups. We will validate the syndrome differentiation using contemporary technologies such as the tongue imaging device and the pulse sphygmograph. An objective diagnosis of the tongue could be achieved with the advancement of artificial intelligence and imaging technology [36,37]. Images of the tongue captured with the tongue diagnostic device will then be processed through an algorithm for comparison and analysis against standard tongue diagnosis images (Figure 2). Furthermore, the development of pulse sphygmographs has enabled the digitalization of pulse assessment. Researchers investigated quantitative analyses of pulse profiles in the time and frequency domains [30]. The development of these quantitative and graphical representations of the pulse profiles in the 6-pulse positions is a significant step toward transforming traditional pulse diagnoses into scientific and objective assessments (Figure 1) [31].

The objective assessment will potentially increase the accuracy of the identification, diagnosis, and prevention of diabetes and prediabetes through a targeted treatment system tailored to the local population.

## Methods

### Study Design and Setting

This nonrandomized, exploratory, observational case-control study integrates the concept of TCM diagnostic methods with contemporary tools in an effort to provide an unbiased perspective on the syndrome differentiation of patients with diabetes and those with prediabetes, and, as a result, provide a guideline for the treatment options specifically targeted at the local population.

**Figure 3:**
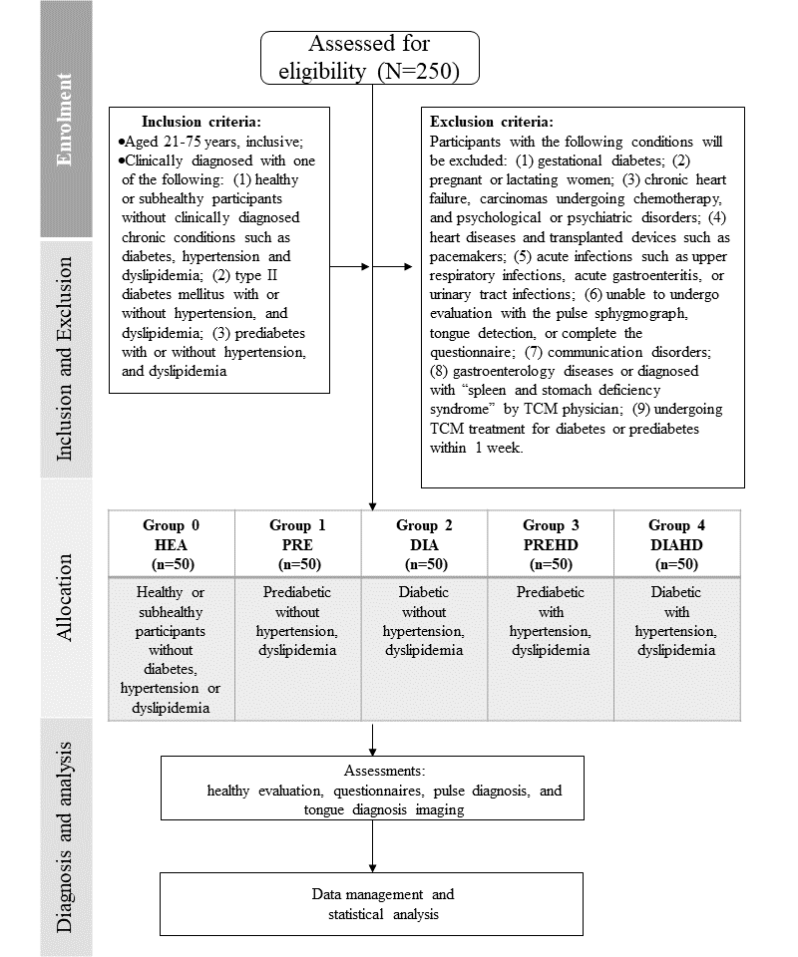
The study flow diagram. DIA: diabetes without hypertension or dyslipidemia; DIAHD: diabetes with hypertension and dyslipidemia; HEA: healthy or subhealthy; PRE: prediabetes without hypertension or dyslipidemia; PREHD: prediabetes with hypertension and dyslipidemia; TCM: traditional Chinese medicine.

The research will be conducted between September 2022 and April 2025 at the Singapore Chung Hwa Medical Institution after obtaining approval from the Parkway Independent Ethics Committee (PIEC; reference: PIEC/2022/003). This study is registered under ClinicalTrials.gov (NCT05563090). Figure 3 depicts the study flow diagram.

### Recruitment of Participants

To ensure a sufficient sample size for statistical analysis of the diabetic and prediabetic syndrome differentiation, we intend to recruit 250 participants who will be equally assigned to one of 5 arms. The groups are as follows: group 0: healthy or subhealthy without diabetes, hypertension, or dyslipidemia; group 1: prediabetes without hypertension or dyslipidemia; group 2: diabetes without hypertension or dyslipidemia; group 3: prediabetes with hypertension and dyslipidemia; and group 4: diabetes with hypertension and dyslipidemia. Primarily, recruitment is conducted through physician referrals, as well as recruitment posters on the institution’s bulletin boards and social media. Participants can register through the digital registration form, while the screening and enrollment will be conducted by the study team. The list of potential participants will be consolidated, and the research assistant will contact the participants to guide them through the informed consent process. Written consent will be collected when informed consent has been explained to the participant in a language they can comprehend. The participants will have the opportunity to decline enrollment at any time, and they are not required to present a justification for doing so. A study ID based on the grouping will be assigned after screening and enrollment according to the inclusion and exclusion criteria. The first 50 participants who qualify for each of the 5 groups and complete the screening process will be included, and recruitment for that particular group will cease once the target number is reached. This ensures that each of the 5 groups has an equal number of participants, allowing for balanced and effective statistical analysis.

Enrollment criteria include: (1) demographic: aged 21-75 years, of any sex or race; (2) clinical conditions include diabetes that have been clinically diagnosed by Western medical professionals (FPG7.0 mmol/L, 2-hour plasma glucose [2hPG]11.1 mmol, or HbA_1c_>6.5%) with or without hypertension and dyslipidemia, prediabetes that have been clinically diagnosed (impaired fasting glucose of FPG 6.1-6.9 mmol/L or impaired glucose tolerance with 2hPG of 7.8-11.0 mmol/L); and (3) control group consists of participants who are healthy or subhealthy and do not have any significant health-related issues, such as clinically diagnosed chronic conditions like diabetes, hypertension, or dyslipidemia. The health evaluation form will be used to confirm that they (1) do not have a family history of diabetes and (2) have not had unusually high FPG levels detected within the last year.

Participants with the following conditions will be excluded from our study: (1) gestational diabetes; (2) pregnant or lactating women; (3) chronic heart failure, carcinomas undergoing chemotherapy, and psychological or psychiatric disorders; (4) heart diseases and transplanted devices such as pacemakers; (5) acute infections such as upper respiratory infections, acute gastroenteritis, or urinary tract infections; (6) unable to undergo evaluation with the pulse sphygmograph, tongue detection, or complete the questionnaire; (7) communication disorders; (8) gastroenterology diseases or diagnosed with “spleen and stomach deficiency syndrome” by TCM physician; and (9) undergoing TCM treatment for diabetes or prediabetes within 1 week.

To protect the confidentiality of personal information, a unique identifier will be used. The principal investigator will maintain a list of study participants and their identification codes.

### Study Procedure and Outcome Measures

Table 3 shows the study schedule for enrollment and assessment, which may take between 0.5 hours and 1 hour in a single visit. The assessment and evaluations will take place in a temperature-controlled environment of 25 °C. The study team has undergone training by the principal investigator on the use of questionnaires, pulse sphygmograph, and tongue diagnostic devices to reduce interphysician variability.

**Table 3:** The study schedule.

		Enrollment	Allocation	Postallocation	
Timepoint		–*t*_1_	0	*t*_1_	*t*_2_
**Registration**					
	Digital registration	✓			
**Enrollment**					
	Informed consent	✓			
	Eligibility screen	✓			
	Allocation		✓		
Intervention				N/A^a^	N/A
**Assessment procedure (0.5-1 hour)**					
	Health evaluation (vital signs, blood sugar levels, BMI, medications, symptoms)			✓	
	Questionnaires (Physical Activity Questionnaire, Sugar Intake, and CCMQ^b^)			✓	
	Pulse diagnosis				✓
	Tongue diagnosis imaging				✓

^a^N/A: data are not applicable or not available.

^b^CCMQ: Constitution in Chinese Medicine Questionnaire.

#### Health Evaluation

Each participant’s age, sex, ethnic race, body weight, height, blood sugar level (including blood glucose in fasting, 2hPG or HbA_1c_ results if available from the patient’s regular blood test report), and health status, including medication prescription, will be recorded. Participants who have been clinically diagnosed as prediabetic or diabetic will be asked to present their blood test results from 3 months prior to or after the assessment date. The study team will contact the participant following the study if the blood test results are not available at the time of assessment. The primary objective of collecting these data is to enable the study team to determine whether the diabetic or prediabetic disorders are under control and if there is a correlation with the syndrome being studied. The information that will be gathered is as follows.

- Vital signs: The Omron Automatic TIC Blood Pressure Monitor HEM-7130 will be used to assess vital signs, including systolic blood pressure, diastolic blood pressure, and pulse rate.
- BMI: After measuring height and weight, the BMI will be calculated and documented.
- Medications: Western and Chinese medications consumed by the participant will be recorded to identify possible interventions and correlations for TCM syndrome differentiation.
- Existing symptoms will be collected to determine the potential correlation with TCM syndrome differentiation.

#### Physical Activity Questionnaire and Sugar Intake

The Physical Activity Questionnaire, adapted and modified from the Singapore Prospective Study Program Physical Activity Questionnaire, will be used to record the level of physical activity during the past 3 months. The Singapore Prospective Study Program Physical Activity Questionnaire evaluates physical activities including transportation, leisure time activities, occupational physical activities, and household activities. This questionnaire was validated against the International Physical Activity Questionnaire in a multiethnic population of Chinese, Malays, and Indians residing in Singapore. It was concluded to show good validity and repeatability for vigorous activities [38].

Metabolic equivalent task (MET) levels will be used to compare the average energy expenditure in each group. The 1 MET unit, which represents the energy expended while sitting quietly, is equivalent to 3.5 ml of O_2_ per kg body weight per minute or 1.2 kcal per minute for a 70-kg individual [39]. Moderate intensity refers to physical activity that is performed between 3 and <6 times the intensity of rest (METs), while vigorous intensity is defined as physical activity that is performed at 6.0 or more METs [38]. The WHO Physical Activity Guidelines (2020) recommend a minimum of 150-300 minutes of moderate-intensity aerobic physical activity per week and engaging in muscle-strengthening activities at moderate or greater intensity on at least 2 days a week to stay physically active [40]. Those who reach a high level of physical activity per week perform vigorous-intensity activity on at least 3 days of 1500 MET-minutes per week, or 7 or more days of activities of a minimum of 3000 MET-minutes. While those with a moderate level of physical activity per week perform 3 or more days of vigorous-intensity activity of at least 20 min/day, or 5 or more days of moderate-intensity activity of at least 30 min/day, or 5 or more days of activities achieving a minimum of at least 600 MET-minutes/week. Participants who do not meet any of the above criteria would be considered inactive.

Additionally, weekly sugary food and beverage consumption will be documented. This will aid in identifying the risk variables involved and the association between the body constitutions and various forms of “*xiao-ke*” based on TCM syndrome differentiation.

#### The Constitution in Chinese Medicine Questionnaire

Based on TCM principles, the Chinese Medicine Questionnaire (CCMQ), which was established in 2009 by the China Association of Chinese Medicine, will be used to evaluate the body constitution of each participant. This questionnaire consists of 60 questions divided into nine categories of body constitution: (1) balanced, (2) qi deficiency, (3) yin deficiency, (4) yang deficiency, (5) phlegm dampness, (6) damp heat, (7) blood stasis, (8) qi stagnation, and (9) inherited special constitution. Each category has 7 or 8 questions. A 5-point Likert scale that quantitatively assesses the body characteristic is adopted, and the original score is obtained by summing the categorized items. The adjusted score is derived by: 100 [(original score – number of questions) / (number of questions 4)]. The scoring algorithm indicates the likelihood of a particular type of body constitution with a higher score. A subscale criterion of at least 30 points will be used to classify the body composition [41]. Table 4 provides the recommended assessing criteria [42].

Lu et al [41] concluded that the CCMQ is capable of measuring the 9 body constitutions with reasonably good construct validity and reliability. Another study reported that the reproducibility using the Spearman correlation coefficient for the subscales ranged from 0.76 to 0.90, indicating good repeatability and quantitativeness [43].

**Table 4:** Assessing criteria of CCMQ^a^.

Adjusted score of balanced constitution	Total adjusted score of 8 unbalanced constitution	Body constitution
≥60	<30	Balanced constitution
≥60	30-39	Balanced constitution but leaning toward the unbalanced constitution with a score ≥40
≥60	≥40	Unbalanced constitution with a score ≥40
<60	≥40	Unbalanced constitution with the highest score
<60	30-39	Prone to an unbalanced constitution with the highest score

^a^CCMQ: Constitution in Chinese Medicine Questionnaire.

#### Pulse Sphygmograph

The Asia Plus Pen Pulse Analysis System Model PPAS-93 (Asia Plus Biotech Co, Ltd) will be used to identify pulse profiles.

The PPAS-93 is a noninvasive device that comprises a high-precision pressure sensor pen and a pulse analyzer. The high-precision pressure sensor pen is a portable handheld pen that digitalizes the biological signal of the radial pressure pulse wave and provides graphical analysis using fast Fourier transformation. This electronic gadget incorporates a high-precision pressure sensor, a filter, an amplifier, and a signal recording card connected to signal analysis software. The sampling rate is 3000 Hz, and the frequency response is between 0.1 and 50 Hz.

With the participant sitting in a relaxed position, the *cun, guan,* and *chi* pulses of each hand will be assessed using the sensor pen with appropriate vertical pressure. The result will be recorded when the best spectrogram displays the highest amplitude. A summation of SE in the 0-10 Hz (SE_0-10Hz_), 10-50 Hz (SE_10-50Hz_), and 13-50 Hz (SE_13-50Hz_) frequency ranges will be obtained. Figure 1 depicts the pulse sphygmograph and its application output.

The time domain analysis examines the amplitude and shape of the arterial waveform such as surging, slippery, fine, and thready [30]. The pulse rate per minute can be determined by the number of waves in 6 seconds multiplied by 10. This will determine the rate such as a rapid or slow pulse. The frequency domain analyzes the variation of SE in the radial pulse waves [30]. The quantitative measures can assess the intensity and density of the SE, providing insight into the deficiency, repletion, coldness, heat, or dampness syndrome. An in-depth examination of both the time and frequency domains will be conducted to deduce the associated syndrome-etiology differentiation of *xiao-ke* (Table 2).

#### Tongue Diagnosis

**Figure 4:**
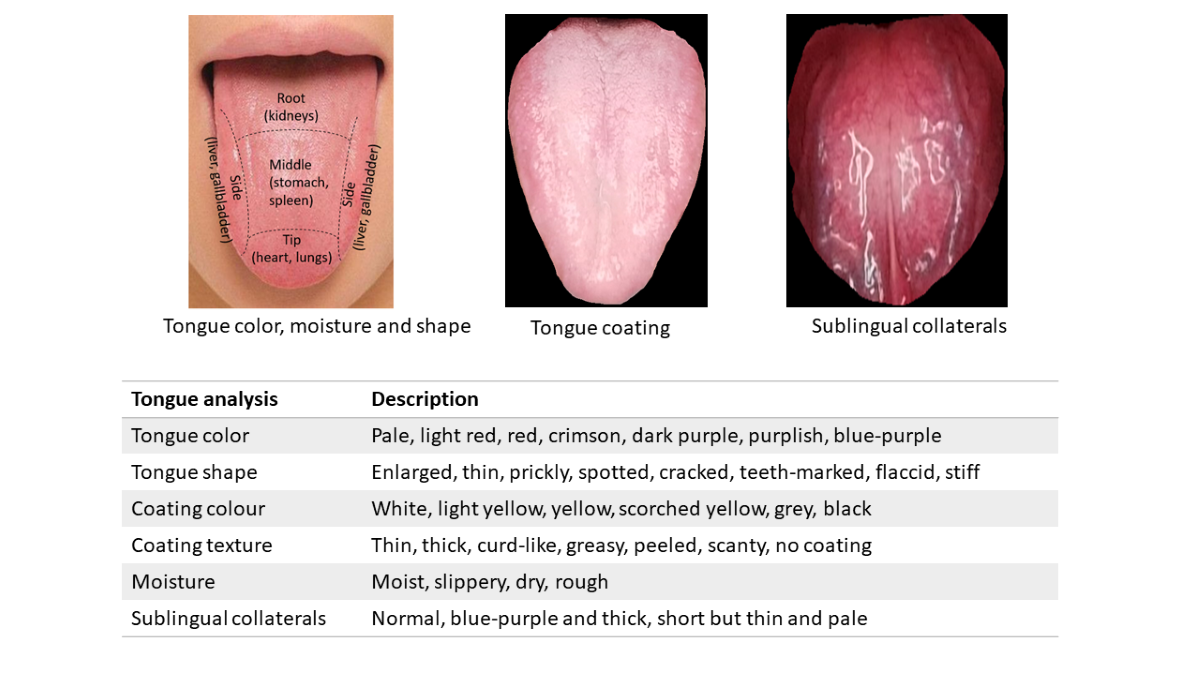
Analytical details of the tongue diagnosis.

**Figure 5:**
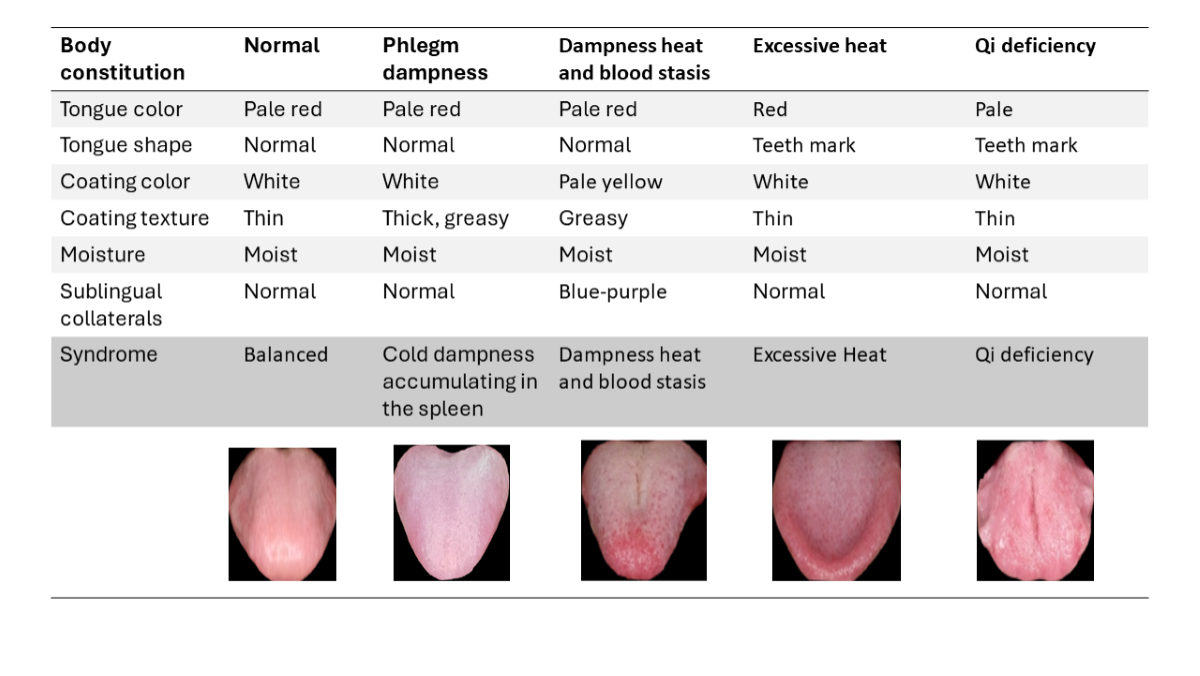
Tongue analysis examples of body constitutions and syndrome differentiation.

The China Artificial Intelligence Health Status Identification System Model YZKJ-SMZY-1AI will be used to image and analyze the participant’s tongue. The noninvasive device captures the image of the tongue through an inbuilt camera supported by light-emitting diode lighting within the device within 15 seconds. A monitor connected to the imaging instrument would display real-time video feedback on the tongue’s position. Using an artificial intelligence system, the photographs of the face, tongue, and sublingual collaterals will be examined and compared with typical images of tongue analysis within 10 seconds. Figure 4 depicts the analytical details of the tongue, including the shape and color of the tongue, the texture and color of the tongue coating, the moisture level of the tongue, and the sublingual collaterals. The body constitution and syndrome differentiation will be analyzed and recorded. Figure 5 provides some examples of body constitutions and syndrome differentiation reported from the system. The results will be reconciled with the pulse diagnosis and CCMQ.

Before evaluation, the participants will be instructed to abstain from tongue scraping, smoking, and consuming caffeinated beverages such as coffee, tea, and cola, as well as dairy products such as milk, yogurt, and cheese, which may alter the tongue diagnosis.

#### Syndrome Differentiation

The syndrome differentiation of *xiao-ke* will be based on the objective assessment results analyzed from CCMQ, tongue, and pulse diagnosis regardless of the participants’ group status. Data collection under standardized conditions using contemporary diagnostic devices is expected to enhance stability and reliability in syndrome differentiation. This approach effectively minimizes bias and eliminates the necessity for blinding in our study design.

### Ethical Considerations

The final clinical trial protocol version 1.8 dated August 1, 2023, was reviewed and approved by the Parkway Independent Ethics Committee (PIEC/2022/003). Important protocol modifications will be communicated to all study staff via emails and meetings after PIEC approval. Informed consent will be obtained from all participants by investigators who are approved by the ethics committee. The participants will be given the right to reject participation at any time. The collected data will be deidentified. Reimbursement in the form of cash vouchers will be provided to the participants upon completion of the study.

### Data Management and Monitoring

Without interference from the study sponsor, the data will be monitored by the principal investigator and the study team. Each participant will be assigned a distinct identifier upon enrollment to guarantee the confidentiality of their information.

The reports generated by the tongue diagnosis imaging device and pulse sphygmograph will be stored as PDFs. Each participant’s information will be documented in an encrypted Microsoft Excel spreadsheet for comparison purposes. No data will be omitted over the duration of the trial. Outliers and missing data will be identified to ensure data quality. All the assessment and evaluation results will be digitized and archived in Microsoft SharePoint with a password-protected database available exclusively to the research staff for at least 6 years following the conclusion of the study before being discarded by the principal investigator.

We anticipated that the study would be completed within the allotted time frame and that there would be no significant participant safety concerns. Consequently, a data monitoring committee and stopping guidelines are unnecessary. Interim analysis may be conducted without stopping the study.

There are no possible risks and side effects from this study as the diagnostic assessments are noninvasive. Hence, adverse events associated with the study are not anticipated.

### Statistical Analyses

The baseline variables (age, sex, height, body weight, BMI, and blood sugar levels), MET-minutes for physical activity, scoring of body constitution, pulse assessments, and tongue analysis results will be compared between the five groups.

Using ANOVA, we will compare the variances across the means of the SE_13-50 Hz_ value from the pulse sphygmograph between each group and the control. Analysis of covariance will also be used to assess the group differences while controlling for potential confounding variables such as gender, age, BMI, physical activity levels, and others. Using the Bonferroni test for post hoc multiple comparisons, the mean values will be compared and corrected. Chi-square tests will also be used to do the comparative analysis of the body constitutions or syndromes between the objective and subjective assessments. All tests will be 2-tailed and *P*<.05 will be considered statistically significant. We will use STATA (standard edition version 18.0; StataCorp LLC) for the analysis.

### Sample Size Calculation

In an earlier 3-arm study to investigate the relationship between dyspepsia and rhinitis conducted by Huang et al [44] with 42 participants assigned to each group, there was a significant difference in the high-frequency pulse SE (SE_13-50Hz_) between the control and experimental groups. Huang et al [45] also compared the radial pulse wave characteristics of hypertensive patients against healthy individuals with 46 patients in each group using the same device. Other investigations using the pulse tonometric device indicated a sample size of 25 with an expectation of 5% type 1 error, 80% power, and 5% drop rates based on the mean and SD derived from a study conducted by Huang et al [46,47]. Although there is limited prior data on the investigation of syndrome differentiation and pulse diagnosis of diabetes and prediabetes, we anticipate that a sample size of 50 in each group will provide adequate power to detect statistical significance.

### Retention of Trial Documents

In a secure storage facility, the principal investigator will maintain all source documentation (including evidence of research eligibility, history and physical findings, and diagnostic test results), as well as ethical review records and other regulatory documentation. The documents should be available for inspection and duplication by authorized parties. The study coordinator will ensure that all documentation is current and up to date.

## Results

Recruitment has been in progress since September 2022.

## Discussion

### Expected Findings

We anticipate that diabetes and prediabetes with or without hypertension and dyslipidemia would have different body constitutions compared to healthy individuals. The prevalence of specific TCM syndrome differentiation may be more common in countries with hot and humid tropical climates, albeit it may differ from the Chinese demographic.

TCM diagnosis is a comprehensive approach that integrates observation, inquiry, pulse palpation, and tongue diagnosis. Finger sensitivity required for palpating radial pulses and precise visual assessment of the tongue are fundamental to accurately detecting variations in the pulse and tongue quality in TCM diagnosis. However, traditional methods of syndrome differentiation are highly subjective and skill dependent. The interpretation of pulse quality and tongue characteristics can differ across practitioners based on their clinical training, experience, and personal bias. This may result in observations lacking clinical significance. To mitigate subjectivity and provide impartial evaluations, we designed this protocol to examine the syndrome differentiation of patients with diabetes and those with prediabetes using contemporary TCM diagnostic technologies. The data collected and evaluated under standardized conditions using these contemporary diagnostic devices are expected to yield stable and unbiased results. This standardized assessment will facilitate more consistent and reliable syndrome differentiation, which is crucial for both clinical practice and research. Thus, our findings may enhance the accuracy of the identification, diagnosis, treatment, and prevention of diabetes and prediabetes through targeted approaches, potentially improving patient outcomes and management effectiveness. Additionally, we will explore and analyze the correlation between TCM syndrome differentiation and WHO diagnosis criteria for diabetes. Establishing the reliability of contemporary TCM diagnostic tools sets the stage for their broader adoption in clinical practice and enables future research into individualized treatment plans based on objective TCM syndrome differentiation. Some of the following study challenges are expected.

#### Sufficient Sample Size

Estimating the sample size for a TCM study, especially when using diagnostic devices investigating syndrome differentiation and pulse diagnosis, can be indeed challenging. There is currently limited preexisting data specifically focusing on the syndrome differentiation and pulse diagnosis in the context of our study. Since this study is a pilot investigation into the TCM syndromes in patients with diabetes and those with prediabetes using digital diagnostic instruments, we used prior studies conducted by Huang et al [44,45] as a reference to estimate the required sample size. These studies used the older model of pulse sphygmograph to investigate the pulse characteristics of chronic conditions such as hypertension, dyspepsia, and rhinitis as well as healthy individuals.

#### Recruiting Eligible Participants

The recruitment of eligible participants is undoubtedly the most crucial, challenging, and unpredictable component of clinical research. Aside from the main strategies such as physician referral, recruitment posters, and social media, publicity can also be disseminated through newspapers, health magazines, or health education to promote awareness and public interest, following the necessary ethical approval. Furthermore, a lack of sufficient knowledge about the study process or clinical trial benefits can be one of the primary barriers to research recruitment. To overcome this barrier, we ensure to provide good communication to the physicians and participants through the designs of advertising posters, health studies, and informed consent. In addition, we reduce the study burden by providing flexibility and accommodation on research visits.

### Conclusions

To the best of our knowledge, this is the first study to use contemporary TCM diagnostic instruments to map expert and empirical knowledge of TCM to its scientific equivalents for the purpose of evaluating the syndrome differentiation of diabetes. In addition to identifying the pulse wave and tongue morphology, both devices are also capable of distinguishing the TCM body constitution and syndrome differentiation through the processing of the identified characteristics by their artificial intelligence algorithm. We will compare and contrast the outcomes of the digital analysis and the CCMQ.

## Abbreviations

2hPG: 2-hour plasma glucose
CCMQ: Constitution in Chinese Medicine Questionnaire
FPG: fasting plasma glucose
HbA1c: glycated hemoglobin A1c
MET: metabolic equivalent task
PIEC: Parkway Independent Ethics Committee
SE: spectral energy
TCM: traditional Chinese medicine
WHO: World Health Organization

## Data Availability

Data sharing is not applicable to this article as no data sets were generated or analyzed during this study.
